# Chemical and transcriptomic diversity do not correlate with ascending levels of social complexity in the insect order Blattodea

**DOI:** 10.1002/ece3.70063

**Published:** 2024-07-31

**Authors:** Marek J. Golian, Daniel A. Friedman, Mark Harrison, Dino P. McMahon, Jan Buellesbach

**Affiliations:** ^1^ Institute for Evolution & Biodiversity University of Münster Münster Germany; ^2^ Department of Entomology & Nematology University of California – Davis Davis California USA; ^3^ Institute of Biology – Zoology, Freie Universität Berlin Berlin Germany; ^4^ Department for Materials and Environment BAM Federal Institute for Materials Research and Testing Berlin Germany

**Keywords:** biosynthesis genes, chemical ecology, cockroaches, cuticular hydrocarbons, eusociality, insect societies, termites, transcriptomes

## Abstract

Eusocial insects, such as ants and termites, are characterized by high levels of coordinated social organization. This is contrasted by solitary insects that display more limited forms of collective behavior. It has been hypothesized that this gradient in sociobehavioral sophistication is positively correlated with chemical profile complexity, due to a potentially increased demand for diversity in chemical communication mechanisms in insects with higher levels of social complexity. However, this claim has rarely been assessed empirically. Here, we compare different levels of chemical and transcriptomic complexity in selected species of the order Blattodea that represent different levels of social organization, from solitary to eusocial. We primarily focus on cuticular hydrocarbon (CHC) complexity, since it has repeatedly been demonstrated that CHCs are key signaling molecules conveying a wide variety of chemical information in solitary as well as eusocial insects. We assessed CHC complexity and divergence between our studied taxa of different social complexity levels as well as the differentiation of their respective repertoires of CHC biosynthesis gene transcripts. Surprisingly, we did not find any consistent pattern of chemical complexity correlating with social complexity, nor did the overall chemical divergence or transcriptomic repertoire of CHC biosynthesis genes reflect on the levels of social organization. Our results challenge the assumption that increasing social complexity is generally reflected in more complex chemical profiles and point toward the need for a more cautious and differentiated view on correlating complexity on a chemical, genetic, and social level.

## Graphical abstract

We assessed the long‐standing hypothesis that chemical profile complexity increases with levels of sociality in the insect order Blattodea investigating solitary as well as eusocial species. Strikingly, we could not confirm any consistent correlative patterns between chemical, transcriptomic, and social complexity. Thus, our results challenge the assumption of a general correlation between increasing social complexity and chemical profile sophistication, which we present here for the first time on a scale uniting both chemical and genetic data in an insect order reflecting all levels of sociality.

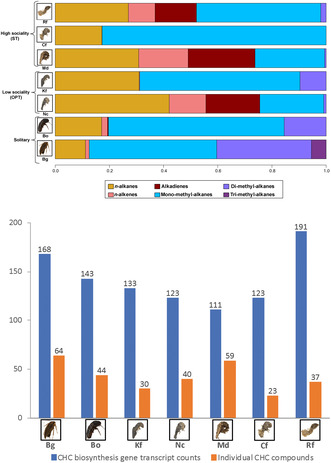

## 1 INTRODUCTION

Insects have exploited chemical signaling as their primary communication mode (Greenfield, 2002; Missbach et al., 2014). Particularly cuticular hydrocarbons (CHCs), non‐polar lipids coating the epicuticle of terrestrial insects, have consistently been demonstrated as pivotal signals and cues in a wide variety of insect chemical communication systems (Blomquist & Bagnères, 2010; Blomquist & Ginzel, 2021). Predominantly, CHCs have been shown to be major signaling molecules for nestmate recognition in eusocial taxa (e.g., Leonhardt et al., 2016; Sprenger & Menzel, 2020) and for sexual and species‐specific signaling mechanisms in solitary taxa (e.g., Chung & Carroll, 2015; Shahandeh et al., 2018). It has been suggested that chemical profiles in eusocial insect societies, with their multiple castes, task allocations, and collective processes, display a higher degree of complexity than in solitary species (Holland & Bloch, 2020; Korb & Thorne, 2017; Kronauer & Libbrecht, 2018; Wittwer et al., 2017). However, there is no consensus as to how to assess, quantify, and compare the degree of chemical profile complexity across different species (Friedman et al., 2020; Holland & Bloch, 2020). Chemical complexity in CHC profiles has previously been assessed as the total number of compounds of a given type or the total ratio of structurally more complex CHC compounds (i.e., unsaturated and methyl‐branched CHCs) versus less complex compounds (i.e., straight‐chain CHCs) (Kather & Martin, 2015; Martin & Drijfhout, 2009). Taking this approach, Kather and Martin (2015) did not find any correlation between CHC diversity and social complexity in a meta‐study comparing chemical profiles in eusocial and solitary Hymenopteran species.

Like the order Hymenoptera (ants, bees, wasps, and sawflies), the order Blattodea encompasses all known levels of social complexity, from solitary cockroaches to obligately eusocial termites. Particularly in termites, which generally lack well‐developed eyes, chemical signaling has been repeatedly demonstrated as the most widespread and dominant form of communication (Bagnères & Hanus, 2015; Van der Meer et al., 1998). In this context, CHCs have been particularly well investigated as fundamental signaling cues for caste differentiation, nestmate recognition, and reproductive status conveyance in termites (e.g., Hoffmann et al., 2014; Liebig et al., 2009; Weil et al., 2009). But in solitary cockroaches as well, CHCs appear to carry out diverse signaling functions, such as kin recognition and aggregation (e.g., Hamilton et al., 2019; Lihoreau & Rivault, 2008; Rivault et al., 1998). To the best of our knowledge, no studies have yet attempted to directly compare CHC diversity across different levels of social complexity within the order Blattodea. In the present study, we compare the levels of CHC profile complexity between representative solitary and social species within the order Blattodea.

Our cockroach study species are *Blatta orientalis* (Blattodea: Blattidae) and *Blattella germanica* (Blattodea: Ectobiidae). The former is known as one of the most common cockroach pest species in temperate regions around the world (Edwards & Short, 1993; Thoms & Robinson, 1986, 1987), whereas the latter is well established in CHC‐based chemical communication research (Fan et al., 2003; Gu et al., 1995; Pei et al., 2019; Rivault et al., 1998). Within termites, although all species are considered eusocial, the level of organization and social complexity mostly divides the different termite taxa into two different life types in terms of colony size, worker sterility, and morphological caste differentiation (Abe, 1987; Korb et al., 2015; Korb & Hartfelder, 2008; Thorne, 1997). One piece life type (OPT) or single‐site termite species are characterized by small colonies and totipotent workers, spending their entire lives nesting and feeding within the same enclosed, wood‐based habitat (Korb & Thorne, 2017; Noirot, 1970; Shellman‐Reeve, 1997). They display an exceptionally flexible caste development, with larval offspring retaining the capability to differentiate into reproductives, alates, or soldiers well into their late instar stages (Korb & Hartfelder, 2008; Noirot, 1985b). This pattern is widely considered to be the ancestral form and is characterized by a low to intermediate form of social complexity (Legendre et al., 2008; Noirot & Pasteels, 1987, 1988). Low social complexity OPT termites are represented in our study by the two species *Kalotermes flavicollis* and *Neotermes castaneus* (Blattodea: Kalotermitidae). Separate life type (ST) or central‐site termite species divide their nesting place from their multiple food sources and are thus characterized by foraging (Abe, 1987; Noirot, 1970; Shellman‐Reeve, 1997). As opposed to OPT termites, ST termites are more constrained in their development due to an early instar separation into a wingless (apterous) line that can further differentiate into permanently sterile soldiers and workers and a nymphal line that eventually develops into sexual alates (Korb & Hartfelder, 2008; Noirot, 1985a; Roisin & Korb, 2010). This pattern characterizes the most socially complex termite species that can reach much larger and more differentiated colonies than OPT termites (Legendre et al., 2008; Noirot & Pasteels, 1987, 1988). *Reticulitermes flavipes* and *Coptotermes formosanus* (Blattodea: Rhinotermitidae) as well as *Mastotermes darwiniensis* (Blattodea: Mastotermitidae) represent ST termites in our study, whereas the latter constitutes a particularly interesting case: The species *M. darwiniensis* is the only extant member of the family Mastotermitidae and phylogenetically represents the most basal termite lineage. However, this species displays all characteristics of ST termites with large colonies, constrained developmental pathways, and a true worker caste (Inward, Vogler, et al., 2007; Krishna et al., 2013). Since the low social complexity OPT has been widely hypothesized to be the ancestral termite state, the clear ST pattern of *M. darwiniensis* as basal and most ancient extant termite lineage represents an unresolved and frequently debated conundrum (Chouvenc et al., 2021; Inward, Beccaloni, et al., 2007; Korb & Thorne, 2017). Through the availability of whole‐genome transcriptomes for our selected study species, we additionally explored CHC biosynthesis gene transcript diversity and correlate it with the different levels of social complexity as well as the respective CHC compound classes they are predominantly associated with. Despite their considerable informative potential on direct links between genetic and CHC variation, studies comparing gene transcript counts or gene expression data with CHC profile complexity are largely lacking so far (Buellesbach et al., 2022; Holze et al., 2021). Most studies on CHC genetics either establish direct functional links between CHC biosynthesis genes and CHC variation through targeted knockdowns (Chung et al., 2014; Dembeck et al., 2015; Sun et al., 2023) or investigate the general genetic architecture underlying CHC compounds as quantitative traits (Buellesbach et al., 2022; Foley & Telonis‐Scott, 2011; Niehuis et al., 2011).

**FIGURE 1:**
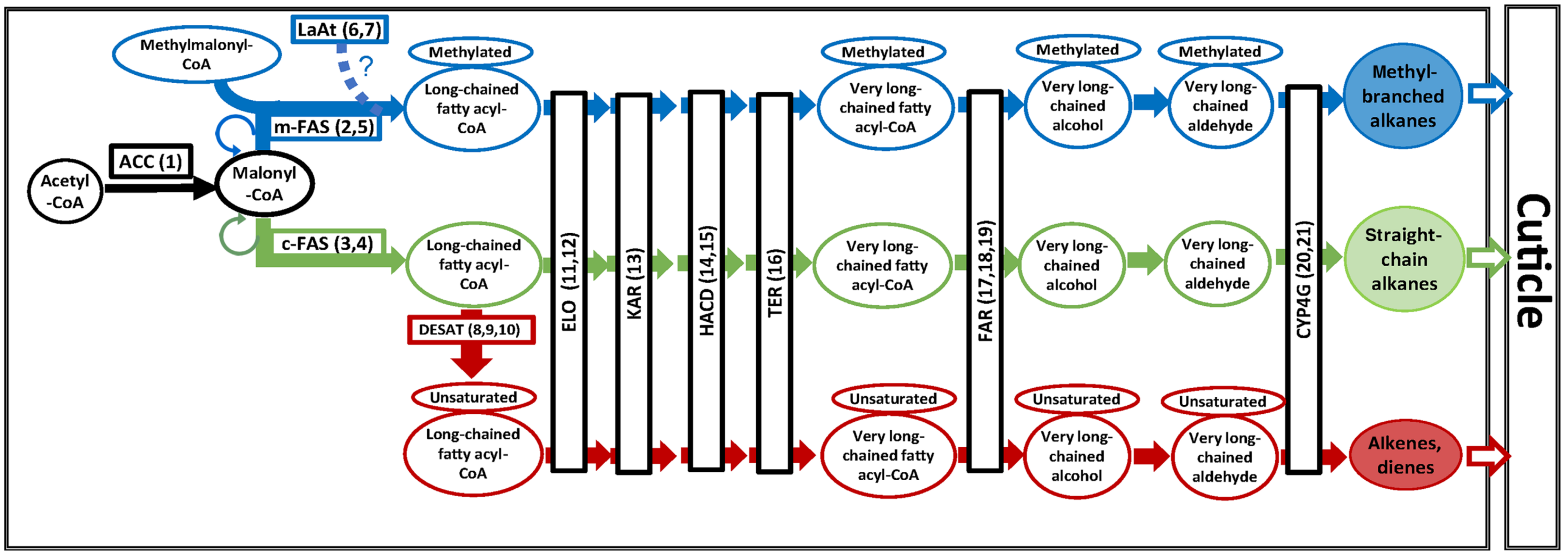
Simplified overview of CHC biosynthesis. The biosynthesis pathway branches at different stages eventually resulting in different CHC compound classes. The main CHC compound classes are methyl‐branched alkanes, straight‐chain alkanes, alkenes, and dienes. Enzyme abbreviations: ACC, Acetyl‐CoA carboxylase; CYP4G, Cytochrome P450 Decarbonylase; ELO, Elongase; FAR, Fatty acyl‐CoA reductase; FAS, Fatty acid synthase (m: microsomal, c: cytosolic); HADC, 3‐hydroxy‐acyl‐CoA‐dehydratase; KAR, 3‐keto acyl‐CoA‐reductase; LaAT, Lipoamide acyltransferase; TER, Trans‐enoyl‐CoA‐reductase. Numbers next to the enzymes correspond to the associated gene transcripts we detected in our tested Blattodea species (compare to Table 1). Adapted from Holze et al. (2021).

We tested the central hypothesis that chemical and social complexity are correlated, and that, concordantly, the genetic repertoire for CHC biosynthesis gene transcripts increases with the level of social complexity. We focused on structurally complex CHC compounds (unsaturated and methyl‐branched) and the candidate genes that potentially play a role in their biosynthesis and variation (mostly desaturases and microsomal fatty acid synthases, see Figure 1 and Holze et al., 2021). Moreover, we constructed a chemical dendrogram based on CHC divergence, compared it to the molecular phylogeny of our study species, and correlated CHC biosynthesis gene transcript counts with the respective CHC compound counts per analyzed species.

## 2 MATERIALS AND METHODS

### 2.1 Tested termite and cockroach species

In a previous study, chemical profiles analyzed among the same taxa were found to be solely discriminable on the species level, rather than on the colony (termites) or population (cockroaches) level (Golian et al., 2022). Therefore, we restricted ourselves to two respective laboratory colonies per termite species and two respective laboratory populations per cockroach species. All species used in this study were maintained in the Federal Institute of Materials Research and Testing (BAM), Berlin. Termite colonies of *R. flavipes*, *C. formosanus*, *K. flavicollis*, and *N. castaneus* were kept in a darkened room at 26°C and 84% humidity, and colonies of *M. darwiniensis* were maintained at 28°C and 83% humidity. All colonies were fed regularly with predecayed birch wood. The cockroaches *B. germanica* and *B. orientalis* were maintained in mixed open rearing boxes in 12‐h light/dark cycles at 26°C and 50% humidity, from the day of egg‐laying until disposal of older adults. Cockroaches were reared on a mixture of 77.0% dog biscuit powder, 19.2% oat flakes, 3.8% brewer's yeast and supplied with water ad libitum and weekly with apple and carrot slices. All cockroaches and termites were freeze‐killed and stored at −20°C until further analysis.

### 2.2 CHC extraction and analysis

To yield comparable amounts of extracts between our cockroaches and termites that vary largely in size, we had to adjust extraction volumes and pool smaller individuals. For this, we used 300 and 3000 μL MS pure hexane (UniSolv, Darmstadt, Germany) on single *B. germanica* and *B. orientalis* individuals, respectively, and 100 μL on pools of three individuals per termite species for extraction. Extraction procedures were then equalized to ensure comparability. Extractions were performed in glass vials (20 mL for cockroaches and 2 mL for termites, Agilent Technologies, Santa Clara, CA, USA) on an orbital shaker (IKA KS 130 Basic, Staufen, Germany) for 10 min. Afterwards, the extract was evaporated under a constant stream of gaseous carbon dioxide (CO_2_). Then, it was resuspended in a 5‐μL hexane solution containing 7.5 ng/μL dodecane (C12) as an internal standard. Three microliters of the resuspended extract was then injected into a gas chromatograph coupled with a tandem mass spectrometer (GC–MS/MS) (GC: 7890B, Triple Quadrupole: 7010B; Agilent Technologies, Waldbronn, Germany) equipped with a fused silica column (DB‐5MS Ultra Inert; 30 m × 250 μm × 0.25 μm; Agilent J&W GC columns, Santa Clara, CA, USA) in splitless mode at a temperature of 300°C with helium used as a carrier gas under constant flow rate of 2.25 mL/min. The temperature program started at 60°C held for 5 min, increasing 20°C/min up to 200°C and then increasing 3°C/min to the final temperature of 325°C, held for 5 min.

Cuticular hydrocarbon (CHC) peak detection, integration, quantification, and identification were all carried out with Quantitative Analysis MassHunter Workstation Software (Version B.09.00/Build 9.0.647.0; Agilent Technologies, Santa Clara, CA, USA). The predefined integrator Agile 2 was used for the peak integration algorithm to allow for maximum flexibility, and quantification was carried out over total ion chromatograms (TICs). All peaks were then additionally checked for correct integration and quantification, and, where necessary, reintegrated manually. CHC compound identification was then carried out based on their characteristic diagnostic ions and retention indices. Analysis was focused exclusively on non‐polar CHC compounds due to their repeatedly demonstrated involvement in chemical signaling in both solitary and eusocial Blattodea (e.g., Hamilton et al., 2019; Hoffmann et al., 2014; Lihoreau & Rivault, 2008). The obtained values for the absolute peak area integrals were standardized by dividing them through the total of all CHC peak area integrals per sample, generating relative proportions for all CHC compounds. These proportions were then summarized for the individual CHC compound classes. Sample sizes for our individual species were *B. germanica*: 9, *B. orientalis*: 11, *C. formosanus*: 5, *K. flavicollis*: 4, *M. darwiniensis*: 4, *N. castaneus*: 5, and *R. flavipes*: 5. We focused on termite workers to obtain the general colony‐specific chemical profiles as the vast majority of individuals constituting the respective colonies are workers (Korb, 2007; Korb & Thorne, 2017; Roisin & Korb, 2010). Moreover, we attempted to render our study comparable to other studies on chemical complexity in eusocial species, also focusing exclusively on profiles obtained from the worker caste (Kather & Martin, 2015; Martin & Drijfhout, 2009). Similarly, we did not discriminate between the sexes to focus on representative species‐specific chemical profiles, with less emphasis on the more subtle sex‐specific differences (Pei et al., 2021).

### 2.3 Comparison of chemical and phylogenetic divergence

To standardize the absolute peak area values for chemical clustering, the normalization method of the function “decostand” of the community ecology R package “vegan” was used (Oksanen et al., 2008), based on the following formula:

$$T_{x,y}=\frac{P_{x,y}}{\sqrt{\sum P_{y^{2}}}}$$

where *T*_*x,y*_ refers to the transformed peak area *x* of individual *y*, *P*_*x,y*_ to the absolute peak area x of individual y, and Σ *P*_*y*_^2^ to the squared sums of all absolute peak areas of individual *y*. This widely applied method for normalizing ecological data was chosen to make the peak areas comparable between our groups, to highlight the relative peak area differences, and to correct for size‐dependent variation. Agglomerative hierarchical cluster analysis (“Unweighted Pair‐Group Method with Arithmetic means”, i.e. UPGMA) was performed with the R package “ape” (Paradis et al., 2004), based on average chemical Manhattan distances reflecting the median CHC divergence separating the different cockroach and termite species. The formula for calculating Manhattan distances is given as follows:

$$\sum \left[Y_{j}-Y_{k}\right]$$

The actual difference between two data points, in this case *Y*_*j*_ and *Y*_*k*_, is used based on the total amount of CHC variation between each compared species pair. In contrast with Euclidean distance where squared differences are used, the Manhattan distance is less prone to be dominated by single large differences within the compared pairs. It has thus been suggested that for multidimensional phenotypes such as CHC profiles, the Manhattan distance metric is the most ecologically meaningful (Oksanen, 2009). The molecular phylogeny was obtained and adapted from the latest published Blattodea phylogeny in He et al. (2021). A Mantel test (Mantel, 1967), conducted with the R package “ade4” (Dray & Dufour, 2007), compared the molecular distances based on the published Blattodea phylogeny with the average Manhattan CHC divergence. The Mantel test was performed five times with 9999 permutations for each single test, and the average probability is presented.

### 2.4 Analysis of transcriptomic gene counts

We retrieved whole‐genome transcriptome sequences based on whole‐body RNA extractions for each of the seven investigated Blattodea species, as described in He et al. (2021). Total RNA was isolated from individuals for all species. Due to the large body size, adult cockroaches were cut into 4–6 parts for separate extraction, followed by re‐pooling. For the extraction itself, samples were suspended in pre‐cooled Trizol (Thermo Fisher Scientific) and homogenized twice at 10 s at 2 M/s with a 5‐mm steel bead (Qiagen) using a tissue homogenizer (MP Biomedicals). Total RNA was isolated with a chloroform extraction, followed by isopropanol precipitation, according to instructions from Trizol. Extracted total RNA was dissolved in RNA storage solution (Ambion) and then incubated with 2 units of TurboDNase (Ambion) for 30 min at 37°C, followed by purification with an RNAeasy Mini kit (Qiagen) according to the manufacturer's instructions. Quantity and quality of RNA were determined by Qubit and Agilent Bioanalyzer 2100, respectively. Following pooling described in the sample collection part, total RNA was used to construct barcoded complementary DNA (cDNA) libraries using a NEXTflex™ Rapid Directional RNA‐Seq Kit (Bioo Scientific). Briefly, messenger RNA (mRNA) was enriched using poly‐A beads from total RNA and subsequently fragmented. First‐ and second‐strand cDNA was synthesized and barcoded with NEXTflex™RNA‐seq Barcode Adapters. The libraries were sequenced on an Illumina NextSeq 500/550 platform at the Berlin Center for Genomics in Biodiversity Research (BeGenDiv). We obtained orthologous sequences of CHC biosynthesis genes, which have been selected according to their demonstrated impact on CHC profiles via targeted knockdown studies (summarized in Holze et al., 2021) from the National Center for Biotechnology Information (NCBI). In order to estimate numbers of transcripts orthologous to these enzymes, we first created Hidden Markov Models (HMMs) for each of their protein sequences. For this, each of the query sequences was blasted against a database of proteomes from 17 insect genomes (*Acyrthosiphon pisum*, *Bemisia tabaci*, *Blattella germanica*, *Clitarchus hookeri*, *Coptotermes formosanus*, *Cryptotermes secundus*, *Diploptera punctata*, *Drosophila melanogaster*, *Glossina morsitans*, *Locusta migratoria*, *Macrotermes natalensis*, *Medauroidea extradentata*, *Musca domestica*, *Periplaneta americana*, *Rhopalosiphum maidis*, *Stomoxys calcitrans*, and *Zootermopsis nevadensis*) with blastp (version 2.7.1+, Camacho et al., 2009). Blast output was filtered to contain only hits with an *e*‐value <1e^−10^ and a minimum sequence identity of 50%. For each query gene, protein sequences of all significant hits were retrieved from the protein database and aligned with PRANK (version v.170427; Löytynoja, 2014) at default settings. Hidden Markov Models (HMMs) were created for each alignment using hmmbuild (version 3.1b2; Wheeler & Eddy, 2013) at default settings. These HMMs were then used to search the proteomes of our seven focal Blattodea species using hmmsearch with a maximum *e*‐value of 1e^−5^. The output of these hmmsearches was then filtered to contain only hits with a score of at least 100. If a transcript appeared in multiple lists, it was attributed to the HMM query for which it received the highest score. Finally, to verify these results, we blasted all transcript sequences against the Swiss‐Prot database (accessed November 2020) with blastp (version 2.7.1+; Altschul et al., 1990). Any transcripts without clear orthology to the gene of interest were then excluded from the transcript counts. We used a generalized linear model (GLM) with Poisson family distribution to compare the variation in transcript counts among the levels of social complexity in our tested species and visualized the results with a heatmap, utilizing the function “heatmap” provided by the R package “stats.” Furthermore, we compared total gene transcript counts with the total number of detected CHC compounds per species with a *χ* ^2^ (chi‐square) test.

## 3 RESULTS

**FIGURE 2:**
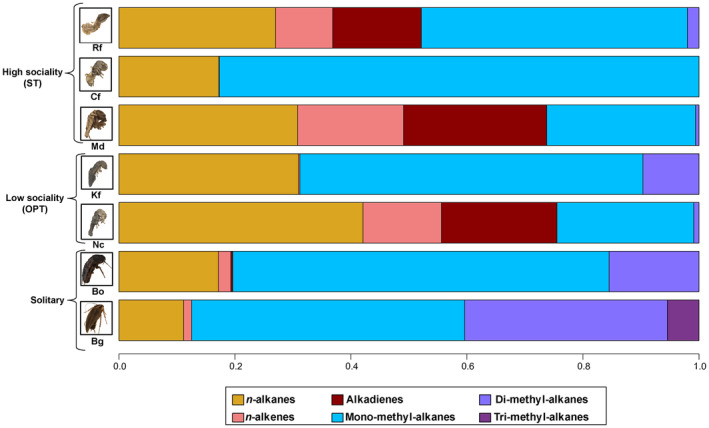
Comparison of average CHC ratios (relative percentages) from the studied representative termite and cockroach species, categorized according to their levels of social complexity. The six major CHC compound classes detected in these species were *n*‐alkanes, *n*‐alkenes, as well as mono‐, di‐, tri‐, and tetra‐methyl branched alkanes and are indicated by different colors. Acronyms for the investigated species are used here and in all subsequent figures as follows: *R. flavipes* (Rf), *C. formosanus* (Cf), *K. flavicollis* (Kf), *N. castaneus* (Nc), *M. darwiniensis* (Md), *B. germanica* (Bg) and *B. orientalis* (Bo). Insect images have been obtained from the Darmstadt Insect Scanner DISC3D (Ströbel et al., 2018) and have been kindly provided by Sebastian Schmelzle.

We identified 134 CHC compounds in total from our representative termite and cockroach species (Table S1). The six major CHC compound classes detected were *n*‐alkanes, *n*‐alkenes, alkadienes as well as mono‐, di‐, and tri‐methyl‐branched alkanes (Figure 2). The relative amounts of the different compound classes varied greatly across all species and not all classes were observed in each species. On average, *n*‐alkanes show higher relative abundances in termites (29.1%) than in cockroaches (15.43%), whereas di‐methyl‐branched alkanes show the reversed pattern with much higher average abundances in cockroaches (25.96%) than in termites (0.03%). Alkadienes were only found in *R. flavipes*, *M. darwiniensis*, and *N. castaneus*, with minimal trace occurrences also present in *B. orientalis*. Generally, mono‐methyl‐branched alkanes were the most abundantly detected compound class across the tested cockroach and termite species; however, they occurred in comparably low quantities in *M. darwiniensis* and *N. castaneus*. Unsaturated compounds, generally considered to be among the structurally more complex CHCs indicating higher chemical complexity together with methyl‐branched alkanes (Kather & Martin, 2015; Martin & Drijfhout, 2009), occur inconsistently in two high (ST) and one low (OPT) social complexity termite species, but only in traces in the cockroaches. However, the most structurally complex methyl‐branched alkanes with three methyl branches occur exclusively in just the cockroach species *B. germanica*. In contrast, the structurally most simple CHC profile, consisting almost exclusively of *n*‐alkanes and mono‐methyl‐branched alkanes, was found in the high social complexity termite *C. formosanus*.

**FIGURE 3:**
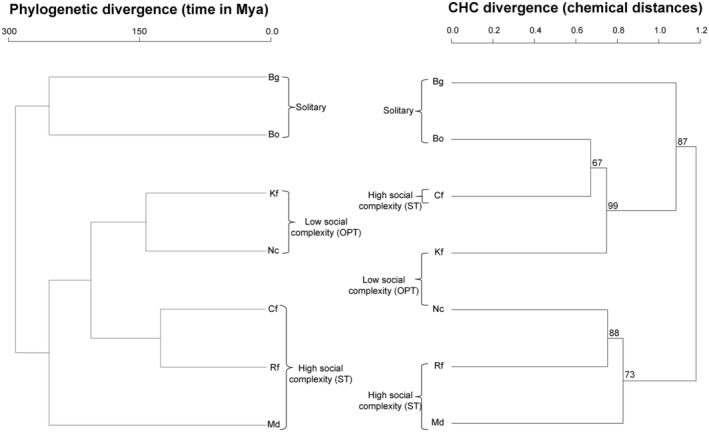
Comparison of the molecular phylogeny (left) and a chemical dendrogram (right) of our Blattodea study species. The molecular phylogeny is adapted from He et al. (2021) and the chemical dendrogram is based on average chemical Manhattan distances reflecting the median CHC divergence separating the different cockroach and termite species. Acronyms as in Figure 2.

The molecular phylogeny of our tested termite and cockroach species mostly mirrors their respective levels of social complexity except for *M. darwiniensis* and *C. formosanus*, which represent the most basal termite groups despite displaying a high level of social complexity (Figure 3). This is not at all reflected in their chemical phylogeny based on the average CHC divergence between the species. Namely, the most highly supported cluster (99 Bootstrap) encompasses a solitary (*B. orientalis*), highly social (*C. formosanus*), and lowly social (*K. flavicollis*) species. Moreover, all levels of social complexity that cluster together in the molecular phylogeny are basically broken off in the chemical phylogeny, with no recognizable pattern. Unsurprisingly, a Mantel test found no significant correlation between the molecular and chemical phylogeny (*r* = 0.3971, *p* = .1291).

**FIGURE 4:**
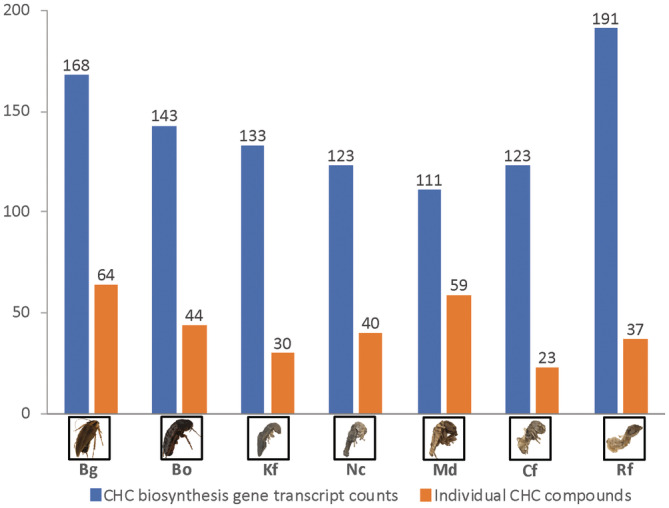
Comparison between counts of CHC biosynthesis gene transcripts (indicated in blue) and total individual CHC compounds (indicated in orange) detected in our representative cockroach and termite species. Correlations between these two metrics were assessed with a *χ* ^2^ (chi‐square) test (*r* = 0.12, *p* = .79). Insect images have been obtained from the Darmstadt Insect Scanner DISC3D (Ströbel et al., 2018) and have been kindly provided by Sebastian Schmelzle.

We also found no significant correlation comparing overall CHC biosynthesis gene transcript counts with the total number of CHC compounds detected in each species (*χ* ^2^ – test, *r* = 0.12, *p* = .79; Figure 4). *R. flavipes* has the highest transcript counts (191) but the third lowest CHC compound count (37), conversely, *M. darwiniensis* has the lowest transcript count (111) but the second highest CHC compound count (59). Again, no trend toward the different levels of social complexity could be detected, which is best exemplified in the three high social complexity termite species, where both the highest (*R. flavipes*) and the lowest (*M. darwiniensis*) numbers of transcripts as well as the second highest (*M. darwiniensis*) and the lowest numbers (*C. formosanus*) of CHC compounds were detected. Across all investigated CHC biosynthesis gene transcripts (Table 1), counts did not vary systematically by social complexity level (*χ* ^2^ = 1.11, df = 1, *p* = .29), which is also apparent in a heatmap representing the individual transcript counts per species normalized by their average relative abundances (Figure 5).

**TABLE 1:** List of 21 genes with a demonstrated function in CHC biosynthesis, for which we could detect transcripts in at least one of our tested cockroach and termite species. The numbers correspond to the position of the respective gene product in the biosynthesis pathway (compare to Figure 1). Gene acronyms, (putative) functions, NCBI (or GenBank when not available in NCBI) IDs, and the taxon where the gene was originally described are indicated along with the detected copy numbers in our tested species. The CHC biosynthesis genes were retrieved from Holze et al. (2021).

#	Gene acronym/(putative) function	NCBI or Gen‐Bank ID	Original taxon	Bg	Bo	Kf	Nc	Md	Cf	Rf
1	*ACC*/Acetyl‐CoA carboxylase	35761	*Drosophila melanogaster* (fruit fly)	8	7	7	5	7	3	7
2	*FASN2*/Fatty acid synthase 2	117361	*Drosophila melanogaster*	16	19	14	6	11	13	17
3	*FASN3*/Fatty acid synthase 3	3355111	*Drosophila melanogaster*	0	0	1	0	2	0	0
4	*BgFas4*/Fatty acid synthase	MK605591.1	*Blatella germanica* (German cockroach)	5	4	2	1	11	2	1
5	*BgFas6*/Fatty acid synthase	MK605593.1	*Blatella germanica*	6	4	1	0	0	1	1
6	*CG5599*/putative NADH dehydrogenase with LaAt activity	32441	*Drosophila melanogaster*	17	6	17	9	7	7	4
7	*CG8680*/putative NADH dehydrogenase with LaAt activity	33744	*Drosophila melanogaster*	1	0	1	1	3	0	1
8	*Desat1*/Desaturase 1	117369	*Drosophila melanogaster*	2	2	0	4	4	1	1
9	*Desat2*/Desaturase 2	41536	*Drosophila melanogaster*	33	23	22	23	10	10	36
10	*Fad2*/Desaturase F	44006	*Drosophila melanogaster*	1	0	0	1	0	1	7
11	*CG18609*/putative Elongase (ELO)	37158	*Drosophila melanogaster*	8	10	10	10	4	7	13
12	*CG9458*/putative Elongase (ELO)	41214	*Drosophila melanogaster*	10	18	10	12	8	6	15
13	*spidey*/3‐keto‐acyl‐CoA‐reductase (KAR)	31703	*Drosophila melanogaster*	3	1	1	1	2	1	1
14	*Hacd1*/3‐hydroxy‐acyl‐CoA‐dehydratase (HADC)	34614	*Drosophila melanogaster*	3	0	2	2	2	2	3
15	*Hacd2*/3‐hydroxy‐acyl‐CoA‐dehydratase (HADC)	34762	*Drosophila melanogaster*	1	2	6	2	2	8	3
16	*Sc2*/trans‐enoyl‐CoA‐reductase (TER)	38457	*Drosophila melanogaster*	4	2	2	2	1	1	2
17	*NlFAR7*/fatty acyl‐CoA reductase (FAR)	MG573162.1	*Nilaparvata lugens* (brown planthopper)	1	1	0	1	1	9	1
18	*NlFAR9*/fatty acyl‐CoA reductase (FAR)	MG573164.1	*Nilaparvata lugens*	1	1	0	0	0	0	0
19	*CG10097*/putative fatty acyl‐CoA reductase (FAR)	3771756	*Drosophila melanogaster*	35	32	28	32	25	38	55
20	*Cyp4g1*/Cytochrome P450‐4 g1	30986	*Drosophila melanogaster*	10	4	4	5	2	9	19
21	*LmCYP4G102*/Cytochrome P450‐4 g1	ANW46746.1	*Locusta migratoria* (grass hopper)	3	7	5	6	9	4	4

**FIGURE 5:**
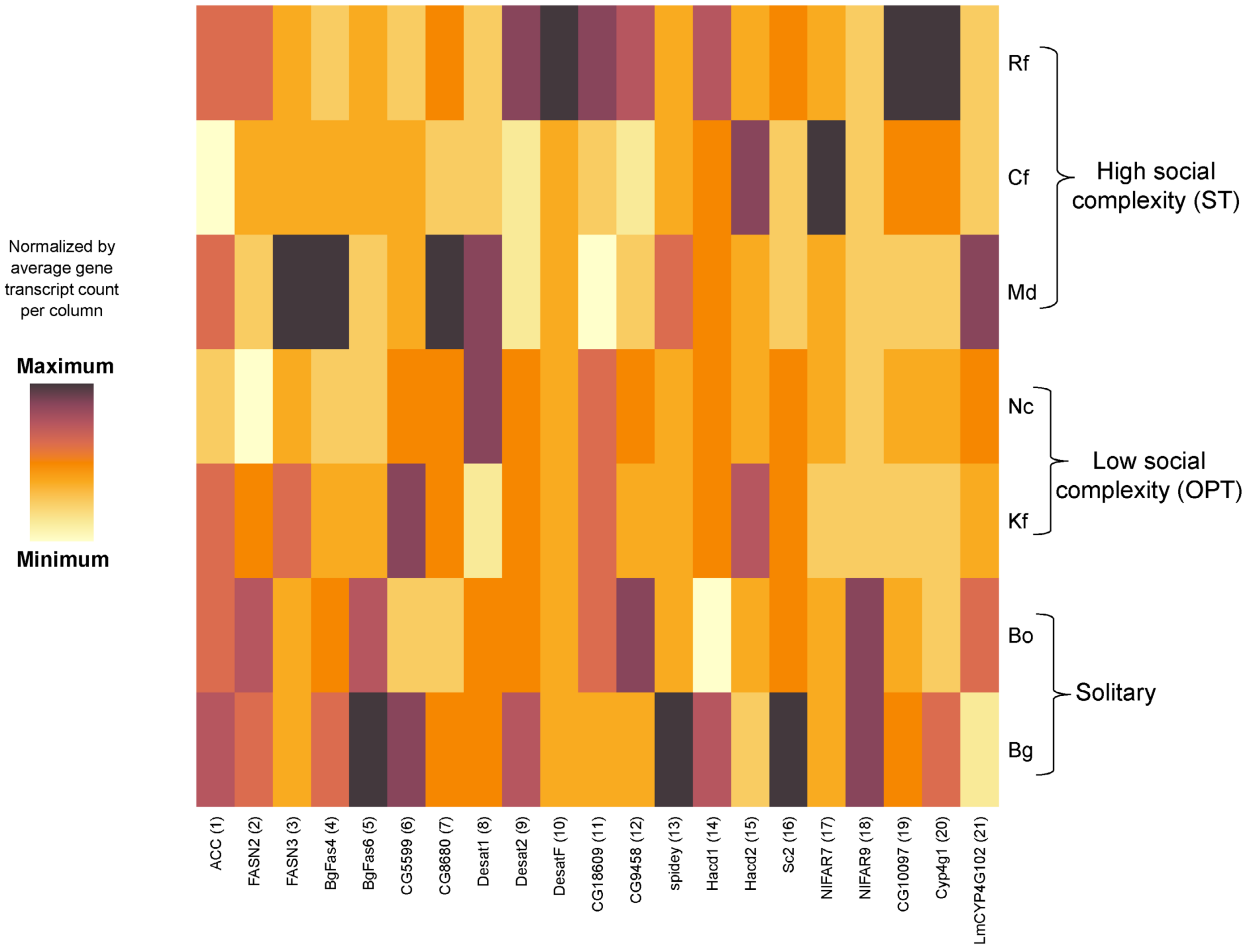
Heatmap normalized by average relative abundances of CHC biosynthesis gene transcript counts (columns) from high (darker colors) to low (lighter colors) grouped by species and their respective level of social complexity. The numbers indicated at the gene transcripts correspond to their respective position in the CHC biosynthesis pathway (see Figure 1 and Table 1).

## 4 DISCUSSION

### 4.1 CHC divergence and variation in relation to social complexity

We compared chemical and associated transcriptomic complexity between Blattodea species with increasing levels of social organization. Overall, we did not find a consistent correlative pattern of CHC‐based chemical complexity paralleling the different levels of social complexity in our representative Blattodea species. These results point to large categorical differences in CHC profiles across species, which appear to be independent from their level of social organization.

CHC divergence based on average chemical distances between the species neither reflects the different levels of complexity nor the phylogenetic divergence within the Blattodea (Figure 3). *M. darwiniensis*, the most basal termite lineage (Inward, Beccaloni, et al., 2007; Inward, Vogler, et al, 2007; Krishna et al., 2013), chemically clusters together with both a high (*R. flavipes*) and a low (*N. castaneus*) social complexity termite, and the solitary cockroach *B. orientalis* clusters with both a high (*C. formosanus*) and a low (*K. flavicollis*) social complexity termite. It has been hypothesized that chemical divergence clearly differing from an established molecular phylogeny indicates selection on chemical profiles for different functions overriding their phylogenetic information (Buellesbach et al., 2013; Marten et al., 2009). Since our chemical divergence does not display any pattern congruent with the social hierarchy of our study species, any assumptions on selection for CHC functions reflective of the species' respective social complexity are rendered highly unlikely. Concerning counts of gene transcripts stemming from orthologs of CHC biosynthesis genes from other insect species, these do neither quantitatively correlate with higher levels of social complexity nor with the total number of CHC compounds detected in each of our study species.

Future work could include more species as transcriptomes, genomes, and chemical profiles become available, and utilize scalable frameworks such as phylogenetically contrasted regression and Bayesian ancestral state reconstruction models (Simon et al., 2019). Additionally, caste‐specific CHC variation in eusocial taxa could be taken into account in future studies as well, potentially adding another layer of complexity, despite the accompanying issues for direct comparability with solitary taxa.

### 4.2 CHC biosynthesis gene transcript variation across the studied Blattodea species

Acetyl‐CoA carboxylase (ACC) catalyzes the first and rate‐limiting step in CHC biosynthesis (Barber et al., 2005, see Figure 1). In each of our analyzed cockroach and termite species, we found several distinct ACC transcripts (ranging from 3 in *C. formosanus* to 8 in *B. germanica*) based on orthology to the ACC gene first described in *Drosophila* (Table 1). This rich abundance of ACC transcripts strengthens the argument for the universality of ACC as fundamental catalyst for the first step in CHC biosynthesis (see also Figure 5).

For fatty acid synthase (FAS) genes, seven of them had already been identified in *Blatella germanica*, with five showing a significant effect on CHC compound quantities upon knockdown (Pei et al., 2019). In our analyzed transcriptomes, we were able to detect transcripts with strong orthology to two of these five FAS genes (BgFas 4 and 6). Transcripts with orthology to *BgFas4* were detectable across all seven species, whereas *BgFas6* transcripts were most abundant in the two cockroaches and only in three of the five analyzed termites (Table 1). Generally, it has been hypothesized that two types of FAS, cytosolic and microsomal, differentially impact CHC biosynthesis, with the former mainly governing the biosynthesis of straight‐chain CHCs, and the latter being more specific for methyl‐branched CHCs (Chung et al., 2014; Wicker‐Thomas et al., 2015, Figure 1). Since we could not detect any transcript copies of *BgFas6* in both *M. darwiniensis* and *N. castaneus*, the two species with the lowest amounts of methyl‐branched alkanes (Figure 2), it is possible that the *BgFas6* transcripts stem from a microsomal FAS associated with the production of methyl‐branched alkanes. Intriguingly, for *N. castaneus*, in addition to showing both the lowest number and proportion of methyl‐branched alkanes, it also shows the lowest transcript copy number of *FASN2* orthologs, an oenocyte‐specifically expressed *Drosophila* gene with a strong effect on methyl‐branched CHCs and thus speculated to be microsomal (Chung et al., 2014; Wicker‐Thomas et al., 2015).

Unsaturated compounds, whose biosynthesis is crucially dependent on desaturases (Coyne et al., 1999; Dallerac et al., 2000; Wicker‐Thomas et al., 1997), occur in each of our studied Blattodea species, most abundantly in *R. flavipes*, *M. darwiniensis*, and *N. castaneus*, intermediately in *B. orientalis* and *B. germanica*, but only in traces in *K. flavicollis* and *C. formosanus* (Figure 2). This partially reflects the respective numbers of transcripts orthologous to the *Drosophila* genes *desat1* and *desat2*, with the former being most abundantly represented in *M. darwiniensis* and *N. castaneus*, and the latter in *R. flavipes* (Table 1).

Genes coding for Cytochrome P450 Decarbonylases of the gene subfamily CYP4G have been shown to govern the final steps in CHC biosynthesis and have thus been suggested to be stable, highly conserved, and particularly vital elements in this pathway (Feyereisen, 2020; Holze et al., 2021, Figure 1). Concordantly, at least one *Cyp4g* gene could be identified in all insect genomes screened to date (Feyereisen, 2020; Qiu et al., 2012). We found transcripts orthologous to the *Drosophila* gene *Cyp4g1* as well as the migratory locust *Locusta migratoria* gene *LmCYP4G102* in all our tested species (Table 1). However, their numbers vary largely with no apparent consistent pattern, hinting at more transcriptomic variety for these vital CHC biosynthesis elements than previously assumed.

However, the assessment of transcript counts as approximation of the actual genomic repertoire for CHC biosynthesis genes naturally has its limits and will remain speculative until targeted knockdown studies confirm the actual functions of the transcripts and their underlying genes. However, analyzing the abundance of unique transcripts per ortholog allows valuable insights into functional diversity potentially exceeding the information contained within whole‐genome sequences (He et al., 2021; Sprenger et al., 2021). Correlating transcriptomic diversity directly with CHC profile variation has been attempted surprisingly rarely despite its potential to approximate the genetic control of CHC variation more accurately, as it has repeatedly been shown that the same genotype can produce different CHC profiles (Holze et al., 2021; Sprenger et al., 2021). Thus, our findings constitute promising first steps for future studies with the potential to corroborate our transcriptomic assessment with target gene counts once further genomic resources become available. For instance, highly contiguous genome assemblies, which are currently scarce in Blattodea, will allow for more accurate gene annotations (Chakraborty et al., 2016; Feldmeyer et al., 2024). This in turn will facilitate more precise predictions of CHC biosynthesis gene counts, especially those that are known to exist in high copy numbers, such as desaturase (DESAT) genes (Helmkampf et al., 2015) and fatty acyl‐coA reductase (FAR) genes (Buellesbach et al., 2022). Lastly, since our knowledge on the genetics of CHC biosynthesis is far from complete (Holze et al., 2021), genes that have not yet been associated with this metabolic pathway might also play a role in governing CHC variation (Moris et al., 2023), and remain yet to be uncovered in the order Blattodea.

### 4.3 CHC‐based communication mechanisms and outlook for future studies

It has long been hypothesized that the complexity of CHC profiles reflects the complexity of the chemically encoded information necessary to maintain more socially complex signaling (Holland & Bloch, 2020; Korb & Thorne, 2017; Kronauer & Libbrecht, 2018). However, how CHC profiles actually encode biologically relevant information such as nestmate affiliation or task allocation has remained largely elusive so far (Buellesbach et al., 2018; Heggeseth et al., 2020; Menzel et al., 2019). Further elucidation on the exact encoding mechanisms in CHC profiles and which compound combinations actually convey chemical information will be instrumental in gaining a better understanding on how insect populations and societies are maintained at different levels of social complexity. Furthermore, although insects generally synthesize the majority of the components in their CHC profiles themselves (Blomquist & Bagnères, 2010; Nelson and Blomquist, 1995), several studies have demonstrated the impact of a variety of biotic and abiotic factors such as diet, microclimate, habitat, and microbiome on CHC profiles as well (Fedina et al., 2012; Rajpurohit et al., 2017; Teseo et al., 2019). Thus, disentangling these factors from the conserved CHC profile functionalities represents an additional challenge in future studies that will nevertheless be indispensable to fully comprehend and explore CHC‐mediated communication mechanisms.

Since we did not find any positive correlations between CHC complexity and levels of social complexity, alternative hypotheses accounting for CHC profile divergence in our tested Blattodea species have to be considered. One suggested hypothesis takes potential conflicts in dominance hierarchies over reproduction into account (“conflict hypothesis,” Gronenberg & Riveros, 2009; O'Donnell et al., 2015). Namely, the hypothesis predicts strong selection on the recognition of individual reproductive states in primitive eusocial taxa or solitary taxa where conflicts over reproduction are common. Therefore, higher levels of CHC complexity in taxa with intermediate to low levels of sociality in accordance with honest signaling in reproductive conflicts would impede cheaters to exploit this communication system (Nehring & Steiger, 2018; Queller & Strassmann, 2009). However, at least for termites, reproductive conflicts have generally been found to be quite low compared to social Hymenoptera (Bai et al., 2022; Hoffmann & Korb, 2011; Sun et al., 2020). Since we also did not find a consistently reversed pattern of higher CHC complexity in low sociality termites or solitary cockroaches as opposed to the more socially complex termites, this hypothesis is unlikely to explain the CHC diversification in our studied taxa.

Another hypothesis claims that CHC complexity constitutes a prerequisite for, rather than a consequence of, social complexity (“precursor hypothesis,” Kather & Martin, 2015; Nehring & Steiger, 2018). This is very evident in Hymenoptera, where all CHC compound classes were apparently already present in early taxa potentially preceding the evolution of eusociality, and extant solitary parasitoid wasps actually possess the most complex CHC profiles (Kather & Martin, 2015). This also fits well with our findings, where it appears unlikely that increasing levels of social complexity have driven the evolution of CHC complexity, at least when regarding the extant state of our analyzed Blattodea taxa.

Future studies should also consider the occurrence of very long‐chained CHC compounds of up to C58 in Blattodea surface profiles, as recently demonstrated with non‐standard analytical methods (Golian et al., 2022). However, neither exact compound quantifications nor identifications (e.g., discrimination between *n*‐alkanes and methyl‐branched alkanes) are so far possible in this higher chain length range extending beyond the CHC compounds traditionally accessed and identified through gas‐chromatographic separation (Bien et al., 2019; Schnapp et al., 2016). Therefore, to include very long‐chain CHCs in future analyses, novel methods need to be established to reliably assess their exact quantities and compound classes. Moreover, despite CHCs constituting the dominant, most investigated compounds in insect chemical communictation (e.g., Blomquist & Bagnères, 2010; Chung & Carroll, 2015; Leonhardt et al., 2016), we cannot exclude the additional potential of non‐CHC compounds also contributing to social complexity signaling (e.g., Hanus et al., 2010; Smith et al., 2016; Steitz et al., 2019). Lastly, CHC metabolic networks could be amenable to various kinds of more elaborate complexity analyses, such as metabolic network pathfinding (Kim et al., 2017), identification of critical connectors (Kim et al., 2019), determination of topological characteristics (Goryashko et al., 2019), and flux balance analysis (Beguerisse‐Díaz et al., 2018). These types of complexity analyses have, to our knowledge, not been attempted so far in this context at all and might largely aid in obtaining a more holistic correlative view on complexity on a chemical, genetic, and social level.

## 5 CONCLUSIONS

Overall, we did not find any consistent patterns linking CHC profile variation, CHC biosynthesis transcriptome diversity, and social complexity across the seven Blattodea species included in our study. This is partially reflective of the results Kather and Martin (2015) obtained in their meta‐analysis of solitary and eusocial Hymenopteran CHC diversity, although this study was lacking the genetic component. This implies that, at least for our representative species spanning different levels of social complexity within the order Blattodea, neither their CHC profiles nor their repertoire of CHC biosynthesis gene transcripts does reflect any social hierarchy or correlate with their social complexity. Concerning the genetic background of CHC biosynthesis, however, it must be taken into account that our general knowledge remains limited and mostly biased towards the model organism *Drosophila* (Holze et al., 2021). Therefore, it is quite possible that more Blattodea‐specific CHC biosynthesis genes exist that have not been functionally characterized yet and have thus eluded our comprehensive gene transcript investigation. Nevertheless, our study challenges the long‐standing assumption of a general correlation between increasing social complexity and chemical profile sophistication for our Blattodea study species. Therefore, we strongly suggest more cautious approaches for assessing, comparing, and interpreting chemical complexity in insects with different levels of social organization.

## AUTHOR CONTRIBUTIONS

**Marek J. Golian:** Data curation (equal); formal analysis (lead); investigation (equal); methodology (lead); validation (equal); writing – review and editing (supporting). **Daniel A. Friedman:** Visualization (supporting); writing – original draft (equal); writing – review and editing (equal). **Mark Harrison:** Data curation (supporting); formal analysis (supporting); investigation (supporting); methodology (supporting); resources (equal); validation (supporting); writing – review and editing (supporting). **Dino P. McMahon:** Funding acquisition (lead); project administration (supporting); resources (equal); supervision (supporting); writing – review and editing (supporting). **Jan Buellesbach:** Conceptualization (lead); data curation (lead); formal analysis (supporting); funding acquisition (supporting); investigation (lead); methodology (supporting); project administration (lead); supervision (lead); validation (lead); visualization (equal); writing – original draft (lead); writing – review and editing (lead).

## CONFLICT OF INTEREST STATEMENT

The authors declare that the research was conducted in the absence of any commercial or financial relationships that could be construed as a potential conflict of interest.

## Supporting information


Table S1.

## Data Availability

All data underlying the present study are available at the dryad data repository under https://datadryad.org/stash/share/czeLte8Tw6y81BBsumFJcVu9EwdN7uNuBHrjuWlIQF8.
