# Supplementary material for: Chemical and transcriptomic diversity do not correlate with ascending levels of social complexity in the insect order Blattodea
Source: Ecol Evol. 2024 Jul 31;14(8):e70063. doi: 10.1002/ece3.70063 (PMC11289792; doi:10.1002/ece3.70063)
Supplement: Supplementary file 1 — Table S1. [file ECE3-14-e70063-s001.docx]

# Appendix

**Tab. S1**: Retention indices (RI), CHC compound identifications and the respective relative quantities as well as standard deviations (in %) for all tested cockroach and termite species. Non-detectable amounts of the respective compounds are indicated by hyphens. Where compound identifications were ambiguous due to multiple possible methyl branch positions based on the detected ion pairs, all possible compound configurations are given. Retention indices we calculated according to the position of detected *n*-alkanes in our samples and, where not available, with a C21-40 *n*-alkane standard run under the same conditions. Please note that for compounds beyond C40, the *n*-alkane positions had to be extrapolated according to the average distances between *n*-alkanes.

| RI | Compound ID | Diagnostic ions | Bg | Bo | Nc | Kf | Md | Cf | Rf |
| --- | --- | --- | --- | --- | --- | --- | --- | --- | --- |
| 1500 | n-C15 | 212 | - | - | 1.04 ± 0.05 | - | - | - | - |
| 1700 | n-C17 | 240 | - | - | 0.06 ± 0.03 | - | 0.01 ± 0 | - | - |
| 1738 | 3-MeC17 | 239; 56; 224 | - | - | 0.14 ± 0.07 | - | - | - | - |
| 2107 | n-C21 | 296 | - | - | 2.33 ± 0.26 | 0.09 ± 0.04 | 0.1 ± 0.03 | - | - |
| 2207 | n-C22 | 310 | - | - | 2.49 ± 0.14 | - | 0.82 ± 0.24 | - | 0.06 ± 0.03 |
| 2270 | 4-MeC22 | 309; 70; 280 | - | - | 1.03 ± 0.15 | - | - | - | - |
| 2280 | C23-ene1 | 322 | - | - | - | - | 0.21 ± 0.06 | - | 1.13 ± 0.46 |
| 2287 | C23-ene2 | 322 | - | - | - | - | 0.06 ± 0.02 | - | 0.07 ± 0.04 |
| 2310 | n-C23 | 324 | 0.07 ± 0.07 | - | 13.48 ± 1.12 | 0.07 ± 0.02 | 7.57 ± 0.71 | - | 7.97 ± 1.38 |
| 2343 | 9-; 11-;13-MeC23 | 323; 140; 224; 168; 196; | 0.01 ± 0.02 | - | 0.83 ± 0.22 | 0.01 ± 0.01 | 0.56 ± 0.18 | - | 0.39 ± 0.18 |
| 2349 | 7-MeC23 | 323; 112; 252 | - | - | - | - | 0.09 ± 0.03 | - | - |
| 2371 | 4-MeC23 | 323; 70; 294 | - | - | 3.45 ± 0.2 | - | 0.89 ± 0.26 | - | 1.46 ± 0.56 |
| 2379 | 7,11-;7,13-DiMeC23 | 309; 112; 266; 182; 196; 210; 168 | - | - | - | - | 0.08 ± 0.02 | - | - |
| 2380 | 3-MeC23 | 323; 56; 308 | 0.02 ± 0.03 | - | 3.24 ± 0.19 | 0.04 ± 0.02 | 0.55 ± 0.13 | - | - |
| 2380 | C24-ene1 | 336 | - | - | - | - | - | - | 0.59 ± 0.25 |
| 2386 | C24-ene2 | 336 | - | - | - | - | 0.09 ± 0.03 | - | - |
| 2394 | C24-ene3 | 336 | - | - | - | - | 0.12 ± 0.04 | - | - |
| 2409 | n-C24 | 338 | - | - | 4.79 ± 0.44 | 1.88 ± 0.39 | 3.33 ± 0.49 | 0.03 ± 0.03 | 5.92 ± 1.62 |
| 2442 | 10-;12-;14-MeC24 | 337; 154; 224; 182; 196; 210; 168 | - | - | 0.11 ± 0.04 | 0.15 ± 0.11 | 0.66 ± 0.16 | 0.1 ± 0.09 | 1.01 ± 0.37 |
| 2463 | C25-ene1 | 350 | - | - | - | - | - | - | 0.22 ± 0.1 |
| 2473 | 4-MeC24 | 337; 70; 308 | - | - | 4.24 ± 1.16 | 4.11 ± 0.16 | 2.63 ± 0.36 | 0.22 ± 0.2 | 11.79 ± 2.02 |
| 2483 | C25-ene2 | 350 | - | - | 3.41 ± 0.62 | - | - | - | 7.84 ± 0.82 |
| 2483 | C25-diene1 | 348 | - | - | - | - | 3.93 ± 0.99 | - | 7.43 ± 1.05 |
| 2480 | 3-MeC24 | 337; 56; 322 | - | - | - | 0.31 ± 0.02 | - | - | - |
| 2484 | C25-ene3 | 350 | - | 0.01 | - | - | 3.84 ± 0.85 | - | - |
| 2492 | C25-ene4 | 350 | - | - | - | - | 1.77 ± 0.36 | - | - |
| 2509 | n-C25 | 352 | 0.32 ± 0.17 | 1.15 ± 0.96 | 11.1 ± 0.87 | 10.67 ± 0.4 | 9.49 ± 0.56 | 5.89 ± 5.17 | 12.91 ± 3.24 |
| 2540 | C25-diene2 | 348 | - | - | - | - | 2.22 ± 0.21 | - | - |
| 2542 | 9-;11-;13-;15-MeC25 (+ unsaturated non-CHC compound) | 351; 140; 252; 168; 224; 196; 224, 168 | 0.12 ± 0.1 | 0.13 ± 0.11 | 3.04 ± 0.89 | 6.51 ± 2.83 | 5.26 ± 0.41 | 11.19 ± 10.08 | 11.86 ± 1.83 |
| 2551 | 7-MeC25 | 351; 112; 280 | 0.03 ± 0.1 | - | - | 3.18 ± 0.24 | 1.42 ± 0.46 | - | 2.39 ± 3.95 |
| 2555 | C25diene3 | 348 | - | - | 6.18 ± 0.93 | - | - | - | 4.41 ± 1.35 |
| 2562 | C25diene4 | 348 | - | - | 2.48 ± 0.33 | - | - | - | - |
| 2557 | 5-MeC25 | 351; 84; 308 | 0.32 ± 0.25 | 1.12 ± 0.76 | - | - | 0.39 ± 0.07 | - | 1.29 ± 0.67 |
| 2577 | 4-MeC25 | 351; 70; 322 | 0.21 ± 0.11 | 6.4 ± 1.81 | - | 5.69 ± 0.4 | 2.84 ± 0.93 | 12.13 ± 1.52 | 4.29 ± 1.46 |
| 2575 | C25diene5 | 348 | - | - | 4.31 ± 0.22 | - | - | - | 3.3 ± 1.99 |
| 2581 | 3-MeC25 | 351; 56; 336 | 0.19 ± 0.11 | 6.28 ± 1.96 | 4.11 ± 0.57 | 5.81 ± 0.58 | 3.94 ± 0.64 | 3.48 ± 2.87 | 6.44 ± 0.62 |
| 2586 | 5,13-DiMeC25 | 337; 84; 322; 210; 196 | 0.11 ± 0.06 | - | - | - | - | - | - |
| 2593 | C26-ene1 | 364 | - | - | - | - | 2.23 ± 0.57 | - | - |
| 2600 | C26-ene2 | 364 | - | - | - | - | 2.52 ± 0.23 | - | - |
| 2608 | n-C26 | 366 | 0.23 ± 0.13 | 0.82 ± 0.47 | - | 5.97 ± 0.27 | 4.51 ± 0.23 | 2.48 ± 1.75 | - |
| 2613 | 3,7-; 3,9-DiMeC25 | 337; 56; 350; 126; 280; 154; 252 | 0.15 ± 0.21 | 0.33 ± 0.36 | - | - | - | - | - |
| 2641 | 12-; 14; 16-MeC26 | 365; 182; 224; 210; 196; 238; 168 | 0.16 ± 0.09 | 3.21 ± 1.15 | - | 8.66 ± 0.76 | 1.89 ± 1.06 | 2.94 ± 2.17 | 0.36 ± 0.14 |
| 2640 | C26diene | 362 | - | - | - | - | 0.53 ± 0.29 | - | - |
| 2645 | 9-MeC26 | 365; 140; 266 | - | - | - | 3.03 ± 0.67 | - | - | - |
| 2651 | 7-MeC26 | 365; 112; 294 | - | - | - | 0.67 ± 0.1 | - | - | - |
| 2651 | 6-MeC26 | 365; 98; 308 | 0.02 ± 0.01 | - | - | - | - | - | - |
| 2656 | 5-MeC26 | 365; 84; 322 | 0.05 ± 0.03 | 0.06 ± 0.03 | - | - | - | - | - |
| 2669 | 4-MeC26 (+ unsaturated non-CHC compound) | 365; 70; 336 | 0.05 ± 0.03 | 0.58 ± 0.24 | - | 5.21 ± 0.16 | - | 5.08 ± 0.98 | 0.7 ± 0.29 |
| 2681 | 3-MeC26 | 365; 56; 350 | 0.07 ± 0.03 | 0.3 ± 0.12 | - | 0.15 ± 0.06 | - | 0.15 ± 0.04 | 0.11 ± 0.04 |
| 2680 | C27-ene1 | 378 | - | - | 2.05 ± 0.61 | - | - | - | - |
| 2684 | C27-ene2 | 378 | - | - | 0.6 ± 0.25 | - | - | - | - |
| 2689 | C27diene1 | 376 | - | - | - | - | 1.48 ± 0.36 | - | - |
| 2694 | C27diene2 | 376 | - | - | - | - | 1.95 ± 0.34 | - | - |
| 2699 | C27-ene3 | 378 | - | - | - | 0.11 ± 0.22 | 2.07 ± 0.36 | - | - |
| 2703 | C27-ene4 | 378 | - | - | - | 0.11 ± 0.22 | 2.09 ± 1.21 | - | - |
| 2711 | n-C27 | 380 | 3.92 ± 0.41 | 11.36 ± 3.14 | 5.13 ± 0.25 | 8.01 ± 0.45 | 4.36 ± 0.48 | 8.05 ± 0.98 | 0.15 ± 0.05 |
| 2744 | 9-; 11-; 13-; 15-MeC27 | 379; 140; 280; 168; 252; 196; 224 | 5.04 ± 0.75 | 26.96 ± 19.32 | - | 13.39 ± 0.75 | 1.01 ± 0.26 | 20.47 ± 3.31 | 0.05 ± 0.03 |
| 2742 | C27diene3 | 376 | - | - | - | - | 5.48 ± 0.28 | - | - |
| 2752 | C28diene1 | 390 | - | - | 0.61 ± 0.14 | - | - | - | - |
| 2756 | C27diene4 | 376 | - | - | 0.69 ± 0.32 | - | 0.48 ± 0.42 | - | 0.11 ± 0.11 |
| 2756 | 7-MeC27 | 379; 112; 308 | - | - | - | 1.26 ± 0.31 | - | 0.22 ± 0.49 | - |
| 2761 | 5-MeC27 | 379; 84; 336 | 3.6 ± 0.47 | 6.37 ± 1.74 | - | - | - | - | - |
| 2760 | C27diene5 | 376 | - | - | - | - | 0.97 ± 0.26 | - | - |
| 2770 | 11,13-; 11,15-; 11,17-; 13,15-; 13,17-; 15,17-DiMeC27 | 365; 168; 266; 210; 224; 238; 196 | 1.67 ± 0.54 | 0.91 ± 0.48 | - | 4.3 ± 0.99 | - | - | - |
| 2775 | 4-MeC27 | 379; 70; 350 | - | - | - | - | 1.01 ± 0.29 | 11.18 ± 3.67 | - |
| 2775 | 9,15-: 9.17-DiMeC27 | 365; 140; 294; 238; 196; 266; 168 | - | - | - | 5.35 ± 0.77 | - | - | - |
| 2779 | C27diene6 | 376 | - | - | - | - | 2.71 ± 0.15 | - | - |
| 2786 | 3-MeC27 | 379; 56; 364 | 3.24 ± 0.31 | 8.59 ± 1.62 | - | 0.85 ± 0.49 | 2.28 ± 0.2 | 6.76 ± 2.96 | - |
| 2784 | C28diene2 | 390 | - | - | - | - | 0.4 ± 0.08 | - | - |
| 2792 | 5,17-DiMeC27 | 365; 84; 350; 266; 168 | 0.58 ± 0.1 | 7.12 ± 1.3 | - | - | - | - | - |
| 2790 | 5,15-DiMeC27 | 365; 84; 350; 238; 196 | 0.12 ± 0.36 | 1.82 ± 1.06 | - | - | 0.48 ± 0.37 | - | - |
| 2791 | 5,9-; 5,11-; 5,13-DiMeC27 | 365; 84; 350; 154; 280; 182; 252; 210; 224 | 2.53 ± 0.57 | - | - | - | - | - | - |
| 2802 | C28-ene | 392 | - | - | - | - | 1.01 ± 0.39 | - | - |
| 2813 | n-C28 | 394 | 2.35 ± 1.3 | 3.3 ± 1.21 | - | 1.31 ± 0.77 | 0.28 ± 0.08 | 0.57 ± 0.43 | - |
| 2814 | 3,9-; 3,11-DiMeC27 | 365; 56; 378; 154, 280; 282; 252 | 2.91 ± 0.61 | 0.06 ± 0.19 | - | - | - | - | - |
| 2816 | 3,7-DiMeC27 | 365; 56; 378; 126; 308 | 1.46 ± 0.58 | 3.8 ± 1.48 | - | - | - | - | - |
| 2841 | 11-; 13-MeC28 | 393; 168; 266; 196; 238 | - | - | - | - | - | 1.85 ± 2.3 | - |
| 2840 | 12-; 14-MeC28 | 393; 182; 252; 210; 224 | 2.66 ± 0.63 | 1.41 ± 0.58 | - | - | - | - | - |
| 2843 | 3,9,11-; 3,9,13-; 3,9,15-; 3,9,17-TriMeC27 | 393; 56; 392; 154; 294; 196; 252; 224; 280; 168 | 0.88 ± 0.39 | - | - | - | - | - | - |
| 2853 | 6-MeC28 | 393; 98; 336 | 0.57 ± 0.22 | 0.07 ± 0.04 | - | - | - | - | - |
| 2858 | 5-MeC28 | 393; 84; 350 | 0.68 ± 0.23 | - | - | - | - | - | - |
| 2864 | C29diene1 | 404 | - | - | - | - | 0.16 ± 0.05 | - | - |
| 2868 | 4-MeC28 | 393; 70; 364 | 2.84 ± 0.74 | 0.27 ± 0.12 | - | - | - | 0.49 ± 0.48 | - |
| 2874 | C29-ene1 | 406 | - | 2.16 ± 1.06 | - | - | - | - | - |
| 2881 | C29-ene2 | 406 | 1.21 ± 0.79 | - | 0.84 ± 0.35 | - | 2.04 ± 2.12 | 0.07 ± 0.09 | - |
| 2880 | C29diene2 | 404 | - | 0.3 ± 0.14 | - | - | - | - | - |
| 2883 | C29diene3 | 404 | - | - | - | - | 4.09 ± 0.28 | - | - |
| 2882 | 3-MeC28 | 393; 56; 378 | 2.27 ± 1.03 | - | - | - | - | - | - |
| 2887 | 5,9-; 5,11-; 5,13-; 5,15-; 5,17-DiMeC28 | 379; 84; 364; 154; 294; 182; 266; 210; 238 | 0.48 ± 0.21 | - | - | - | - | - | - |
| 2894 | C29-ene3 | 406 | - | - | - | - | 0.12 ± 0.02 | - | - |
| 2897 | 4,10-; 4,12-; 4,14-; 4,16-; 4,18-; 4,20-DiMeC28 | 379; 70; 378; 168; 280; 196; 252; 224; 308; 140 | 0.83 ± 0.61 | - | - | - | - | - | - |
| 2909 | n-C29 | 408 | 4.26 ± 0.9 | 0.51 ± 0.24 | 1.64 ± 0.36 | 2.96 ± 1.53 | 0.34 ± 0.11 | 0.23 ± 0.17 | - |
| 2920 | 3,7-; 3,9-; 3,11-DiMeC28 | 479; 56; 392; 126; 322; 154; 294; 182; 266 | 1.48 ± 0.54 | - | - | - | - | - | - |
| 2940 | 11-; 13-; 15-MeC29 | 407; 168; 280; 196; 252; 224 | 7.25 ± 0.51 | 1.87 ± 0.88 | - | - | 0.07 ± 0.02 | 5.96 ± 8.04 | - |
| 2953 | 7-; 9-MeC29 | 407; 112; 336; 140; 308 | 3.26 ± 1.16 | 0.64 ± 0.3 | - | - | - | - | - |
| 2964 | 5-MeC29 | 407; 84; 364 | 4.4 ± 4.32 | - | - | - | 0.02 ± 0.02 | - | - |
| 2979 | 11,17-; 11,19-; 11,21-; 13,17-; 13,19-DiMeC29 | 393; 168; 294; 266; 196; 322; 140; | 2.14 ± 1.19 | - | - | - | - | - | - |
| 2983 | 7,11-DiMeC29 | 393; 112; 350; 182; 280 | 1.91 ± 0.85 | - | - | - | - | - | - |
| 2985 | 7,17-DiMeC29 | 393; 266; 196 | - | 0.14 ± 0.09 | - | - | - | - | - |
| 2986 | 3-MeC29 | 407; 56; 392 | 4.8 ± 0.67 | 0.08 ± 0.03 | - | 0.14 ± 0.09 | - | 0.47 ± 0.47 | - |
| 2994 | 5,9-; 5,11-; 5,13-DiMeC29 | 393; 84; 378; 154; 308; 182; 280; 210; 252 | 1.98 ± 0.48 | 0.1 ± 0.06 | 0.02 ± 0.04 | - | - | - | - |
| 3013 | 3,7-DiMeC29 | 393; 56; 406; 126; 336 | - | 0.45 ± 0.23 | - | - | - | - | - |
| 3019 | 3,9-; 3,11-DiMeC29 | 393; 56; 406; 154; 308; 182; 280 | 6.44 ± 0.76 | - | 0.03 ± 0.07 | - | - | - | - |
| 3031 | 3,7-DiMeC29 | 393; 56; 406; 126; 336 | 0.63 ± 0.31 | - | - | - | - | - | - |
| 3041 | 3,7,11-; 3,7,13-; 3,7,15-; 3,7,17-; 3,9,11-; 3,9,13-; 3,9,15-; 3,9,17-TriMeC29 | 421; 56; 420; 126; 350; 196; 280; 224; 252; 280; 196; 154; 322; | 4.4 ± 0.68 | - | - | - | - | - | - |
| 3061 | 14,18-; 14-22-DiMeC30 | 407; 210; 266; 280; 196; 336; 140; | 0.86 ± 0.43 | - | - | - | - | - | - |
| 3063 | 4-MeC30 | 421; 70; 392 | 0.34 ± 0.24 | - | - | - | - | - | - |
| 3075 | C31ene | 434 | 0.18 ± 0.11 | 0.01 ± 0.02 | - | - | - | - | - |
| 3076 | C31diene | 432 | - | - | - | - | 0.25 ± 0.13 | - | - |
| 3080 | 5,9-; 5,11-; 5,13-; 5,15-DiMeC30 | 407; 84; 392; 154; 322; 182; 294; 210; 266; 238 | 0.17 ± 0.09 | 0.03 ± 0.1 | - | - | - | - | - |
| 3088 | 4,8-; 4,10-; 4,12-DiMeC30 | 407; 70; 406; 140; 336; 168; 308; 196; 280 | 2.39 ± 0.86 | 0.01 ± 0.04 | - | - | - | - | - |
| 3102 | 3,9-; 3,11-DiMeC30 | 407; 56; 420; 154; 322; 182; 294 | 0.59 ± 0.41 | - | - | - | - | - | - |
| 3112 | 4,8,12-; 4,8,14-; 4,8,16-TriMeC30 (+ acetic acid) | 435; 70; 420; 140, 350; 210; 280; 238, 252; 266; 224 | 0.16 ± 0.12 | - | - | - | - | - | - |
| 3130 | 11-; 13-; 15-MeC31 | 435; 168; 308; 196; 280; 224; 252 | 3.13 ± 0.76 | - | - | - | - | - | - |
| 3154 | 13,17-; 15,17-DiMeC31 | 421; 196; 294; 266; 224 | 2.16 ± 1 | 0.11 ± 0.38 | - | - | - | - | - |
| 3178 | 5,9-;5,11-; 5,13-; 5,15-;5,17-DiMeC31 | 421; 84; 406; 154; 336; 182; 308; 210; 280; 238; 252; 266; 224 | 1.83 ± 0.66 | - | - | - | - | - | - |
| 3203 | 3,9-; 3,11-; 3,13-; 3,15-DiMeC31 | 421; 56; 434; 154; 336; 182; 308; 210; 280; 238; 252 | 0.52 ± 0.25 | 0.01 ± 0.03 | 0.01 ± 0.03 | - | 0.02 ± 0.02 | - | - |
| 3477 | C35-ene | 490 | - | - | - | - | 0.12 ± 0.04 | - | - |
| 3531 | 11-MeC35 | 491; 168; 364 | - | - | - | - | - | - | 0.55 ± 0.31 |
| 3629 | 12-MeC36 | 505; 182; 364 | - | - | - | - | - | - | 0.09 ± 0.06 |
| 3729 | 11-; 13-MeC37 | 519; 168; 392; 196; 364 | - | - | 0.05 ± 0.05 | - | - | - | 2.28 ± 1.11 |
| 3751 | 11,15-DiMeC37 | 505; 168; 406; 238; 336 | - | - | - | - | - | - | 0.45 ± 0.23 |
| 3774 | 5,15-; 5,17-DiMeC37 | 505; 84; 490; 236; 336; 266; 308 | - | - | - | - | - | - | 0.65 ± 0.33 |
| 3840 | C39diene | 544 | - | - | 0.28 ± 0.1 | - | - | - | - |
| 3876 | C39-ene | 546 | - | - | 2.24 ± 0.18 | - | - | - | - |
| 3927 | 11-;13-;15-MeC39 | 547; 168; 420; 168; 392; 224; 364 | - | - | 2.74 ± 0.41 | - | 0.06 ± 0.02 | - | 0.8 ± 0.37 |
| 3949 | 11,15-DiMeC39 | 533; 168; 434; 238; 364 | - | - | - | - | - | - | 0.57 ± 0.26 |
| 3953 | 13,17-DiMeC39 | 533; 196; 406; 266; 336 | - | - | 0.24 ± 0.06 | - | - | - | - |
| 3973 | 5,15-; 5,17-DiMeC39 | 533; 84, 518; 238; 364; 266; 336 | - | - | - | - | - | - | 0.32 ± 0.16 |
| 4054 | C41diene | 572 | - | - | 5.32 ± 0.18 | - | - | - | - |
| 4074 | C41-ene | 574 | - | - | 1.92 ± 0.5 | - | - | - | - |
| 4084 | C41-ene | 574 | - | - | 2.49 ± 0.36 | - | - | - | - |
| 4143 | 11-; 13-; 15-; 17-MeC41 | 575; 168; 448; 196; 420; 224, 392; 252; 364 | 1.72 ± 1.2 | 0.52 ± 0.37 | 0.62 ± 0.23 | - | 0.11 ± 0.03 | - | 0.02 ± 0.04 |
| 4173 | 13,15-; 13,17-DiMeC41 | 561; 196; 434; 238; 392; 266; 364 | 0.99 ± 1.52 | 0.6 ± 1.98 | 0.58 ± 0.15 | - | - | - | - |
|  |  |  |  |  |  |  |  |  |  |
